# Masked Tardive Dyskinesia in the Coronavirus Disease 2019 Era

**DOI:** 10.7759/cureus.16999

**Published:** 2021-08-08

**Authors:** Christopher Laboe, Ankit Jain, Hector Cardiel Sam

**Affiliations:** 1 Pyschiatry, Pennsylvania State University College of Medicine, Hershey, USA; 2 Adult Psychiatry, Pennsylvania State Health Milton S. Hershey Medical Center, Hershey, USA; 3 Child and Adolescent Psychiatry, Pennsylvania State Health Milton S. Hershey Medical Center, Hershey, USA

**Keywords:** mph, attention-deficit/hyperactivity disorder (adhd), methylphenidate, adolescent, masked

## Abstract

The universal donning of masks during the coronavirus disease 2019 pandemic led to a decreased ability to read people’s facial expressions. In this context, it is prudent to think about how diagnoses can be missed or worsened by the practice of mandatory masking. Here, we report the case of an adolescent white male who presented with new-onset agitation, akathisia, and orofacial dyskinesia in the context of chronic stimulant use for the treatment of attention-deficit hyperactivity disorder and antipsychotic use for oppositional defiant disorder.

## Introduction

The year 2020 marked a period when the entire world was on lockdown as the coronavirus disease 2019 (COVID-19) pandemic led to social isolation and avoidance of public spaces. Globally, many regions required the indoor use of a mask. People struggled due to spending more time at home and decreased support from important individuals in their lives. The universal donning of masks led to a decreased ability to read people’s facial expressions [1]. In this context, it is prudent to think about how diagnoses can be missed or worsened by the practice of mandatory masking [2]. Since the pandemic, children have experienced increased depression, anxiety, and stress that may lead to increased suicidal behavior. Attention-deficit hyperactivity disorder (ADHD) is a known psychiatric disorder of childhood. It is considered to be a neurodevelopmental disorder that may persist from childhood into adulthood. In childhood, it is associated with several symptoms such as inattention, hyperactivity, and impulsivity. Symptoms may change or evolve as a person gets older with an increased risk of developing psychiatric comorbidities such as depression, anxiety, and substance addiction [2]. Pharmacotherapy is the most common intervention for ADHD. Stimulant medications are highly effective and are considered the gold standard for treating the inattention, impulsivity, and excessive motoric activity associated with ADHD. Methylphenidate (MPH) and amphetamine-based stimulants are now available in different forms including longer-acting, once-daily, and shorter-acting divided dosing schedules. The mechanism of action of MPH has been well studied; however, stimulant use with compulsory masking leading to undiagnosed tardive dyskinesia (TD) is a new phenomenon. Here, we report the case of an adolescent white male who presented with new-onset agitation, akathisia, and orofacial dyskinesia in the context of chronic stimulant use for the treatment of ADHD and antipsychotic use for oppositional defiant disorder (ODD).

## Case presentation

A 14-year-old white male with a history of ADHD and ODD presented with worsening agitation over two weeks. The agitation stemmed from an argument with his parents regarding failing grades and a refusal to attend virtual school in the context of the COVID-19 pandemic. While in the inpatient unit, the patient described feelings of restlessness that were distressing to him. In addition, perioral TD was observed in the inpatient unit. The presence of a mask may have abetted the observance of TD during the patient’s treatment course. The patient was noted to have an Abnormal Involuntary Movement Scale (AIMS) score of 13. His electrolyte levels, complete blood count, magnetic resonance imaging/electroencephalography, and other basic lab tests were normal. The patient’s home medications included ziprasidone 40 mg, venlafaxine 112.5 mg, MPH 80 mg, and guanfacine 4 mg daily to address impulse control and mood symptoms. His family revealed that he had been prescribed numerous medications with poor efficacy including Adderall XR, fluoxetine, risperidone, aripiprazole, citalopram, and escitalopram. The patient was on antipsychotics in the context of ODD and to address irritation and aggression.

Approximately three years ago, he was reported to be taking MPH 20 mg, MPH patch 30 mg, aripiprazole 2 mg, and guanfacine 2 mg ER, which led to the development of extrapyramidal side effects (EPS). Although the patient was given benztropine as needed in the emergency room for EPS, it was unclear if it was effective. During the hospital admission, a few weeks before psychiatric hospitalization, the dose of ziprasidone was decreased to 20 mg as he continued to have notable EPS including akathisia and tremors, but there were no improvements. According to his family, the patient was easily agitated and defiant at home. History obtained from both the patient and through our extensive correspondence with the family revealed virtual schooling and dissatisfaction with home life as psychosocial stressors.

We initially targeted symptoms of restlessness and TD with 2 mg twice daily propranolol. Propranolol was tolerated well and there were some improvements noted in akathisia but no observable improvement in dyskinetic movements. Because of the lack of efficacy noted by the family and the patient, ziprasidone was gradually weaned off. We hoped that tapering off ziprasidone for a few days would result in improvement in EPS but there was no improvement in dyskinesia following discontinuation. Subsequently, it was postulated that the dyskinetic movements were possibly exacerbated by the MPH, so it was discontinued. Two days after discontinuation of MPH, the patient’s akathisia and TD had completely resolved, with a repeat AIMS score of 0. The patient was started on atomoxetine and titrated up on atomoxetine to 60v mg for his ADHD which was well tolerated. The patient was subsequently discharged home, with improvement in agitation and resolution of restlessness and dyskinetic movements.

## Discussion

Although the mechanism of action of MPH remains poorly understood, the drug has long been thought to block the reuptake of dopamine in the brain [3]. Multiple hypotheses have emerged to describe the cause of dyskinesia while taking a stimulant in ADHD, including the increase in drug serum levels leading to the overstimulation of dopamine receptors, hypersensitivity of basal ganglia receptors, and/or children exhibiting basal ganglia damage similar to that in Parkinson’s disease [4]. MPH is a stimulant that enhances the effects of catecholamine agonists by increasing the availability of dopamine and norepinephrine and has been shown to be effective in the treatment of ADHD. Recent positron emission tomography imaging studies have shown an uptick in dopamine transporter density because of chronic stimulant use that can result in an overall dopamine deficit [5]. The question of psychostimulants, including MPH, causing dyskinesia and akathisia has been explored; however, evidence suggesting causality is limited. Multiple case reports suggest dyskinesia with stimulant use in the setting of ADHD. A case report of a seven-year-old boy on 1 mg risperidone and 10 mg MPH for ADHD has been reported. In our understanding, the pharmacodynamics of the drug changes very little with age so we were able to refer to this paper. The patient was exhibiting unilateral dystonia resulting in repeated right-sided mandibular dislocation that completely resolved upon discontinuation of both medications and prescription of oxazepam [6]. In our case, the patient reported restlessness causing discomfort and dyskinesia (including tongue and extremities) in the absence of symptoms of ADHD or worsening of any of his other psychiatric comorbidities. In this patient, the diagnosis of TD became challenging as a result of mask-wearing during the COVID-19 pandemic. Other conditions have worsened as a result of compulsory masking including posttraumatic stress disorder, anxiety, depression, and obsessive-compulsive disorder [7,8].

Regarding the patient’s dyskinetic movements, one explanation could be a possible interaction with other D2 receptor blockers such as the ziprasidone; however, tapering off and discontinuation of the medication resulted in no change or resolution of EPS. Another possible explanation for the emergence of TD shortly after initiation of MPH may lie in MPH’s well-documented effect on increasing dopamine levels in the striatum [9]. This effect on dopamine levels is evident at the early stages of MPH therapy, a timeline that is consistent with the onset of symptoms in our patient. It is possible that with reduced dopamine activity alone, there was a profound reduction in involuntary movements.

## Conclusions

There is a known correlation between MPH and EPS in patients with ADHD. Studying the relationship between MPH and EPS can yield important information regarding the presentation and outcomes of EPS in adolescents taking stimulants. In addition, mandatory masking has increased the complexity of diagnosing TD due to face covering. In cases such as ours, the adolescent may not immediately experience prominent TD and other symptoms that readily alert those around the patient to the bothersome, adverse effects of EPS. This case report highlights the importance of continuing to evaluate the risk of EPS when prescribing stimulant agents in the setting of the COVID-19 pandemic.
